# Hepatitis B and C Infections in Patients With Prolonged Hemodialysis Secondary to Chronic Renal Failure

**DOI:** 10.7759/cureus.10905

**Published:** 2020-10-12

**Authors:** Wajeeha Elahi, Ameen Zubair Syed, Fahad Nasim, Adnan Anwar, Atif A Hashmi

**Affiliations:** 1 Nephrology, Tabba Kidney Institute, Karachi, PAK; 2 Nephrology, Liaquat National Hospital and Medical College, Karachi, PAK; 3 Physiology, Al-Tibri Medical College, Karachi, PAK; 4 Stereotactic Radiosurgery/Radiation Oncology, Al-Tibri Medical College, Karachi, PAK; 5 Pathology, Liaquat National Hospital and Medical College, Karachi, PAK

**Keywords:** hepatitis b, hepatitis c, hemodialysis, chronic renal failure, chronic kidney disease

## Abstract

Objective

To determine the frequency of hepatitis B and C in patients with prolonged hemodialysis suffering from chronic renal failure.

Methods

A retrospective study was conducted from January to August 2017 at Tabba Kidney Institute, Karachi. A total of 255 patients on hemodialysis were included in the study by using convenient sampling technique. All the relevant data such as gender, age, duration of hemodialysis and presence of hepatitis B and C were recorded. Data were analyzed using Statistical Package for Social Sciences, version 21 (IBM Corp., Armonk, NY) while binary logistic regression was applied to develop a risk assessment model for the study outcomes.

Results

The study results showed that 134 (52.5%) patients were on hemodialysis for five years or more, 173 (67.8%) of them suffered from hepatitis B while 124 (48.6%) of them suffered from hepatitis C. The study results further revealed that after controlling for the effects of age and gender, the duration of hemodialysis was significantly associated with both hepatitis B (AOR 1.917, 95% CI 1.111-3.306, p=0.019) and hepatitis C (AOR 2.323, 95% CI 1.395-3.870, p=0.001) among the patients studied.

Conclusion

The study concluded that longer duration of hemodialysis in patients with chronic renal failure was significantly associated with both hepatitis B and hepatitis C infections in the study population. A myriad of patient and environmental factors can contribute to this finding in patients with chronic renal insufficiency. Therefore, periodic monitoring of liver function tests and routine surveillance for viral hepatitis can help establish an early diagnosis of infection, potentially improving patient outcomes improving patient outcome.

## Introduction

Viral hepatitis is a leading cause of health loss as well as deaths worldwide. Its burden and relative rank had been raised to seventh position in 2013 compared with 10th to 12th rank in 1990 [1]. The Global Health Sector Strategy (GHSS) on viral hepatitis and World Health Assembly in 2016, set a goal for elimination of hepatitis as a public health threat by 2030 so that the death rate will be reduced which is accountable due to hepatitis B virus (HBV) and hepatitis C virus (HCV) infections [2]. Furthermore, after the introduction of new and improved antiviral therapy and direct-acting antiviral (DAA) therapy for chronic HCV, its efficacy and uptake; there is a great opportunity to improve public health by reducing liver-related mortality [3]. 

Chronic renal failure (CRF) patient’s treatment with maintenance invasive hemodialysis (HD) therapy, badly affects the immune system of the patients. The procedure itself reduces the cellular immunity of HD patients. Several changes occur in normal physiology of these patients such as functions of leukocytes, neutrophils and monocytes and as well as natural killer cells become affected [4]. Additionally, HD therapy also leads to defective proliferation and down-regulation of the phosphorylation pathways of T-lymphocytes [5]. Due to weak immune system, the patients receiving hemodialysis (HD) therapy are prone to acquire blood-borne viral infections like hepatitis B virus (HBV), hepatitis C virus (HCV) and human immunodeficiency virus (HIV) as compared to those with the strong immune system [6]. These viral infections alone or with co-infections may induce hepatic inflammation, leading to hepatic cirrhosis and eventually, hepatocellular carcinoma [7]. The prevalence of HBV infection in dialysis centers varies according to the country. It ranges between 1.2% and 6.6% in developed countries and 1.3% to 14.6% in developing countries [8]. Similarly, the percentage of HCV infection ranges from 4.7% to 41.9% in developing countries and 1.4% to 28.3% in developed countries [9].

Factors associated with viral hepatitis in HD patients include a high incidence of HBV and HCV infections in the general population, lack of standard prevention measures and improper or no vaccination, no screening of patients for HBV and HCV before HD therapy, no proper arrangement for disinfecting dialysis machines as well as the spread of infection from one patient to another, recurrent blood transfusions, and long-term exposure to infectious agents through the extracorporeal circulation [10]. HCV and HBV infections are reported usually due to patient to patient transmission within HD units [11]. 

This is very important for health professionals and policymakers to give importance to frequency of blood-borne viruses in HD units and to identify the major risk factors so that they can operate the system more efficiently. This will ultimately decrease the rate of viral infections; lower the rates of morbidity and mortality. The goal of the present study was to investigate the prevalence of viral infections HBV and HCV, in patients with the end-stage renal failure on hemodialysis in our setting.

## Materials and methods

This was a retrospective study conducted from January to August 2017 at Tabba Kidney Institute, Karachi, Pakistan. Keeping the percentage frequency of the study outcome as 50% for the most liberal estimate, with 95% confidence level and 6.5% precision, the minimum sample size was calculated to be 228 participants. Against the calculated sample size, a total of 255 patients were included in the study by using the convenient sampling technique. Patients aged more than 18 years who were on hemodialysis for more than six months were included in the study. Patients with multi-organ failure, with illnesses other than kidney disease, and those who refused to give informed consent were excluded from the study. Later, participants were explained about the research and informed consent was obtained.

All the relevant data such as gender, age, duration of hemodialysis, and the presence of hepatitis B and C were recorded. The patients were divided into two groups based on the duration of hemodialysis: (i) less than five years duration and (ii) five years or greater duration.

Data were analyzed on the Statistical Package for Social Sciences (SPSS) version 21 (IBM Corp., Armonk, NY). Descriptive analysis was performed by generating means and standard deviations for continuous variables and frequencies and percentages for categorical variables. Binary logistic regression was applied to develop a risk assessment model for the study outcomes while keeping the significance level at 0.05.

## Results

A total of 255 patients were included in the study. The mean age of the study participants was 49.43 ± 14.61 years. 134 (52.5%) of the patients were above 49 years of age. Almost one-half were female, 129 (50.6%). The mean duration of the hemodialysis was 6.50 ± 5.33 years. 134 (52.5%) of the patients were on hemodialysis for more than five years. 173 (67.8%) of them suffered from hepatitis B while 124 (48.6%) of them suffered from hepatitis C (Table 1).

**Table 1:** Demographic and clinical characteristics of patients

Variables (n=255)	Mean ± SD/frequency (%)
Age (Years)	49.43±14.61
Age Groups	
Up to 49 Years	134 (52.5)
50 or Above Years	121 (47.5)
Gender	
Male	126 (49.4)
Female	129 (50.6)
Hemodialysis Duration (Years)	6.50±5.33
Hemodialysis Duration Groups	
Less than 5 Years	121 (47.5)
5 or More Years	134 (52.5)
Hepatitis B	
Yes	173 (67.8)
No	82 (32.2)
Hepatitis C	
Yes	124 (48.6)
No	131 (51.4)

The study results further showed that after controlling for the effects of age and gender, the duration of hemodialysis was significantly associated with both hepatitis B (AOR1.917, 95% CI 1.111-3.306, p=0.019) and hepatitis C (AOR 2.323, 95% CI 1.395-3.870, p=0.001) where patients who were on hemodialysis for five years or more had significantly higher odds of having hepatitis B and hepatitis C than those who were on hemodialysis for less than five years (Table 2).

**Table 2:** Adjusted odds ratios of association between hemodialysis duration and hepatitis B and C

Variables (n=255)		Adjusted Odds Ratio	95% CI		p-value
			Lower	Upper	
		Hepatitis B			
Hemodialysis Duration (Years)	5 or More	1.917	1.111	3.306	0.019
		Hepatitis C			
Hemodialysis Duration (Years)	5 or More	2.323	1.395	3.87	0.001

## Discussion

Chronic kidney disease (CKD) is linked with kidney damage and decrease glomerular filtration rate (GFR) for almost three months. CKD badly affects the quality of life, enhance the health care expenditures, rate of morbidity and mortality, and lead to premature death [12]. Long-standing CKD results in end-stage kidney disease (ESKD). In ESKD, uremic waste products are retained in the body and require renal replacement therapies, such as HD and kidney transplantation [13]. HD therapy is utilized not only in those patients who have an acute illness and need short-term dialysis for days to weeks so that kidney will resume its function, and also on those patients who are critically ill with advanced CKD and/or ESKD and necessitate long-term or permanent renal replacement therapy [14].

It has been observed that viral infections are common in chronic hemodialysis (CHD) patients, particularly the viral hepatitis B and C, and HIV infections. Its prevalence differs from country to country and between HD centers.

Present study was conducted in Karachi, Pakistan; in this study, we found that the prevalence of HBV was high in HD patients with a duration more than five years, and that there is a significant positive association between HBV positivity and duration of dialysis. Our results are distressingly high as compare to previously mentioned results from other previous studies conducted in Pakistan. A study conducted in Karachi, Pakistan showed 10.2% of HD patients [15], another study conducted in Islamabad, Pakistan depicted 12.5% of HD patients [16], one study from Quetta showed 16.1% of HD patients found to be positive for HBV [17], these results were in contrast to 6% HBV positivity in general population of Pakistan [18]. All these studies also showed that the duration of dialysis was significantly correlated with positivity of HBV in HD patients similar to our findings. WHO's consideration for countries to be high endemic region of HBV is almost 5% carrier rate [19]. However, the prevalence of hepatitis B in Pakistan is around 4% to 6% in general population [20]. Because there is large pool of delta positive cases in some regions of Sind, Punjab and Baluchistan [20-22] and also a low EPI coverage of hepatitis B vaccine, the chances for the spread of viral infection are high [23]. Hence, regarding our findings, we can postulate that this high burden of HBV in HD patients is probably due to already high burden of HBV in general population and particularly immune-compromised chronic kidney disease patients too. The other important factor is the contamination of dialysis machines after reuse and lack of following standard operating procedures regarding the cleanliness of equipment. 

The prevalence of HCV is 48.6% in HD patients with a duration of more than five years in the present study. The positivity of HCV in HD was significantly associated with the duration of dialysis. Our results are comparable with other previous studies conducted worldwide and in Pakistan. A study conducted in Karachi showed that the HCV was positive in 20% of HD therapy patients [13]. Similarly, another study from Quetta Pakistan showed that 43.2% of HD patients were positive for HCV [15]. These results are comparable with our findings. A multicenter study conducted in Saudi Arabia involving 22 HD centers and including 1147 patients found 68% of HD were positive for HCV ranges from 14.5% to 94.7% [24]. This was the highest prevalence observed in the world. They also depicted that this was significantly associated with duration of dialysis. The most important risk factor they describe was a lack of adherence to standard universal infection precautions.

The viral hepatitis infections are major health issues in both developed and developing countries because of being the leading cause of health loss and deaths in patients receiving HD treatment. The progressive risk to develop either cirrhosis or hepatocellular carcinoma after viral hepatitis is very high in HD patients. This is high time for healthcare professionals and policymakers to take urgent actions against this endemic condition. HD centers must adopt internationally accepted immunization standards and infection control policies, in order to minimize the burden of HBV and HCV in patients already burdened by their advanced kidney disease.

We view our study with a few limitations. First, we utilized convenient sampling for data collection, which introduces bias by multiple factors that cannot be controlled. Confounding factors that could not be controlled in our study include lack of information regarding HBsAb status and history of vaccination before the diagnosis of chronic hepatitis B. Centre for Disease Control and Prevention (CDC) recommends hepatitis B and C screening at least once in a lifetime for all adults aged 18 years and older, except in settings where the prevalence of HBV and HCV infection is less than 0.1% [25]. Unfortunately, in Pakistan, no such screening is performed routinely. In fact, in certain rural and urban areas, screening for blood donors is also not performed and syringes are reused that ultimately leads to a high disease burden. 

## Conclusions

Hemodialysis is among the major factors that cause viral infections including HBV, HCV, or HIV, this is because patients on hemodialysis can catch an infection through blood transfusion, dialysis machines, instruments, or other contaminated equipment. There is an immense need to control the spread of viral infections through several means like increased public awareness, patients screening before dialysis, vaccinations, and health education programs for both health care providers and patients.
